# Perceived Stress and Coping Strategies in Relation to Body Mass Index: Cross-Sectional Study of 12,045 Japanese Men and Women

**DOI:** 10.1371/journal.pone.0118105

**Published:** 2015-02-12

**Authors:** Chisato Shimanoe, Megumi Hara, Yuichiro Nishida, Hinako Nanri, Yasuko Otsuka, Kazuyo Nakamura, Yasuki Higaki, Takeshi Imaizumi, Naoto Taguchi, Tatsuhiko Sakamoto, Mikako Horita, Koichi Shinchi, Keitaro Tanaka

**Affiliations:** 1 Department of Preventive Medicine, Faculty of Medicine, Saga University, Saga, Japan; 2 Department of Public Health, Showa University School of Medicine, Shinagawa-ku, Tokyo, Japan; 3 St. Mary’s College Faculty of Nursing, Kurume, Japan; 4 Laboratory of Exercise Physiology, Faculty of Sports and Health Science, Fukuoka University, Fukuoka, Japan; 5 Chikushi Office for Health, Human Services and Environmental Issues, Fukuoka Prefectural Government, Onojo, Japan; 6 Division of International Health and Nursing, Faculty of Medicine, Saga University, Saga, Japan; Kyushu University Faculty of Medical Science, JAPAN

## Abstract

**Background:**

Accumulated evidence suggests a weak positive relationship between psychosocial stress and body mass index (BMI), but little is known about stress coping strategies and BMI.

**Objective:**

We aimed to examine if perceived stress and coping strategies are related to BMI, with any of their mutual interactions on BMI.

**Methods:**

This cross-sectional study included 5,063 men and 6,982 women aged 40-69 years. A self-administered questionnaire ascertained perceived stress and 5 items of coping strategies (emotion expression, emotional support seeking, positive reappraisal, problem solving, and disengagement). Analyses were performed by gender with adjustment for age, socioeconomic status, and lifestyle factors.

**Results:**

No significant associations were detected between perceived stress and BMI in either men (*P*_trend_ = 0.09) or women (*P*_trend_ = 0.58). In men, however, ‘disengagement’ showed an inverse association with BMI (*P*_trend_ < 0.001), and ‘positive reappraisal’ and ‘problem solving’ revealed a positive association with BMI (*P*_trend_ = 0.04 and 0.007, respectively) even after controlling for perceived stress. A possible interaction between perceived stress and ‘disengagement’ on BMI was found in men (*P*_interaction_ = 0.027); the inverse association between ‘disengagement’ and BMI was more evident in higher levels of stress (*β* = −0.13, *P*_trend_ = 0.21 in low; *β* = −0.22, *P*_trend_ = 0.01 in medium; and *β* = −0.24, *P*_trend_ = 0.06 in high). In men, ‘disengagement’ was inversely associated with overweight/obesity (odds ratio 0.79, 95% confidential interval 0.67-0.95), and “positive reappraisal” was positively associated with it (1.25, 1.02-1.54).

**Conclusions:**

Coping strategies may have an important role in developing overweight/obesity, particularly in men.

## Introduction

Psychosocial factors have been independently associated with cardiovascular diseases (CVD) [1] although they have shown weaker associations than other established risk factors such as smoking and obesity [2]. Psychosocial stress may lead to CVD possibly through developing obesity[3], and thus its association with indices of adiposity including the body mass index (BMI) have been investigated [4,5]. In a recent meta-analysis of 14 longitudinal studies on psychosocial stress and adiposity (evaluated mostly with BMI), the majority (69%) of correlation coefficients (r) revealed no significant relationships, followed by significant positive associations (25%) and significant inverse ones (6%), with an overall very weak positive association (combined r = 0.014, 95% confidence interval 0.002–0.025) [4]. Additional two longitudinal studies suggested a positive association [6] or no association [7] whereas two large cross-sectional studies demonstrated a weak positive association [5,8]. Accordingly, the overall evidence appears to support a positive association between psychosocial stress and BMI although the magnitude of the association is very weak.

The individuals’ efforts to manage stressors, coping strategies [9], have been conceptualized as moderators of the association between psychosocial stress and health-related outcomes [10]. Excess cortisol secretion is considered to be one of the mechanisms for the relationship between psychosocial stress and weight gain [11], and one recent study indicated that the lack of a coping strategy (i.e., each of self-protection, problem engagement, and seeking social support) was associated with elevated glucocorticoids [12,13]. We therefore hypothesized that coping strategies may be potential predictors of obesity as well as moderators of the link between stress and obesity. To our knowledge, only one study suggested an inverse association between a coping strategy (i.e., confrontive coping) and BMI in 180 women [14], and little is known about whether coping strategies moderate the association between psychosocial stress and BMI, or vice versa. The impact of gender difference on the association between coping strategies and BMI may also be of concern due to the paucity of such data.

We conducted a cross-sectional study of a middle-aged and older general Japanese population in order to examine if perceived stress and coping strategies are related to BMI, with any mutual interactions and gender differences in their possible relations to BMI.

## Methods

### Study population

The Japan Multi-Institutional Collaborative Cohort Study (J-MICC Study) was initiated in 2005 with the aim of obtaining fundamental data for the prevention of life-style related diseases, mainly cancer, according to genetic traits [15,16]. As part of participants in the baseline survey of the J-MICC Study between 2005 and 2007, we enrolled 12,078 men and women who were aged 40–69 years and resident in Saga city, Japan [17–19]. Ten subjects withdrew later, and the remaining 12,068 subjects constituted the study population of this cross-sectional study. Baseline measures included height (cm), weight (kg), socioeconomic status (SES: occupation, working hours, and years of schooling), lifestyle factors (alcohol consumption, smoking, physical activity, sleeping hours, and energy intake), and psychosocial factors (perceived stress and coping strategies). The subjects completed a self-administered questionnaire, and any missing or inconsistent answers were checked by a research nurse at the survey location.

All subjects gave written informed consent, and the study protocol was approved by the ethics committees at both Saga University Faculty of Medicine and Nagoya University Graduate School of Medicine.

### Perceived stress and Coping strategies

Perceived mental stress in daily life was assessed by a single question [19,20], “How much stress did you feel during the last year?” Four response categories were given as: 1) “I felt much stress”, 2) “I felt moderate stress”, 3) “I felt little stress”, and 4) “I felt no stress at all.” The perceived stress level was regarded as high for the response category 1); medium for the category 2); and low for the categories 3) and 4) combined. We assigned scores of 1–3 to low, medium, and high levels of perceived stress, respectively. The weighted *κ* for this question in a 1-year reproducibility survey on a random sample (n = 431) was 0.55 [19], which was analogous to that reported in the INTERHEART study (Cohen’s *κ =* 0.5) [1].

For coping strategies, we used 5 items selected from a dispositional version of the General Coping Questionnaire [21,22] or the brief COPE [23]. These coping strategies were evaluated as follows. After a query, “How do you cope with various problems and unfavorable events you experience in daily life?”, subjects were requested to answer the frequency (four response categories: “seldom”, “sometimes”, “often”, and “very often”) of each of the following coping strategies: 1) “I express my negative feelings and thoughts” (termed ‘emotion expression’), 2) “I consult with someone close and ask him/her for encouragement” (termed ‘emotional support seeking’ [ESS]), 3) “I try to interpret the problem in a favorable way” (termed ‘positive reappraisal’), 4) “I try hard to solve the problem” (termed ‘problem solving’), and 5) “I let the problem take its own course” (termed ‘disengagement’). The level of each coping strategy was classified as low for the frequency of “seldom”, medium for “sometimes”, and high for “often” or “very often”. The weighted *κ* in the reproducibility survey ranged from 0.30 to 0.55 for these coping strategies [19].

### Covariates

Occupation, working hours (including those for housework), and years of schooling were considered as indicators of SES. Subjects’ occupation at the baseline survey was asked in an open-ended manner, and their responses were classified according to the International Standard Classification of Occupations, 2008 (ISCO−08) into 4 categories: 0–2 (armed forces occupations, managers, and professionals); 3–5 (technicians, associate professionals, clerical support workers, and service and sales workers); 6–9 (skilled agricultural, forestry and fishery workers, craft and related trades workers, plant and machine operators, assemblers, and elementary occupations); and unemployed subjects including retired persons and housewives. Years of schooling were estimated based on the school from which subjects graduated.

Lifestyle factors included drinking and smoking habits, physical activity, sleeping hours, and energy intake. Total ethanol consumption (g/day) was estimated from reported consumption frequencies and amounts of 6 types of alcoholic beverages. Physical activity was assessed with a single-axis accelerometer [24] (Kenz Lifecorder Ex; Suzuken Co., Ltd., Nagoya, Japan) worn by subjects for 10 days after the baseline survey. The physical activity level was calculated as total energy expenditure (kcal/day) divided by basal metabolic rate (kcal/day); the former was estimated from the accelerometer as average daily energy expenditure (excluding the initial 3 days), and the latter was defined as basal metabolism standard [25] × body surface area [26] × 24 hours. Total energy intake was estimated using the information from a validated short food frequency questionnaire [27,28] and standard tables of food composition in Japan (Fifth Revised Edition) [29] as previously described [18].

### Statistical analysis

From among the 12,068 participants in the baseline survey, we excluded 23 subjects who had missing data on perceived stress or BMI (n = 19), or a dietary energy intake greater than 4000 kcal/day (n = 4). Consequently, 12,045 subjects (5,063 men and 6,982 women) remained for this study. All analyses were performed separately for men and women with the SAS statistical software package (Ver. 9.3 for Windows; SAS Institute, Cary, NC, USA).

The differences of baseline characteristics by BMI levels (<25 and ≥25 kg/m^2^) in men or women were tested by t tests (for continuous variables) or chi-square tests (for categorical variables). The associations of perceived stress and 5 coping strategies (each 3 categories [low, medium, and high] converted to an ordinal variable) with BMI (kg/m^2^) were evaluated by simple and multiple liner regression analyses. Covariates for 1) age (years), 2) SES (occupation [4 categories by ISCO−08 as described above], working hours [4 categories: < 5, ≥ 5−< 7, ≥ 7−< 9, and ≥ 9], and years of schooling [5 categories: ≤ 9, 12, 14, 16, and ≥ 17]), 3) lifestyle factors (drinking [5 categories: never, former, and current drinker consuming 0.1–22.9, 23.0–45.9, and ≥ 46 g ethanol/day], smoking [5 categories: never, former, and current smoker consuming 1–19, 20–39, and ≥ 40 cigarettes/day], physical activity level [continuous], sleeping hours [4 categories: <6, ≥ 6−< 7, ≥ 7−< 8, and ≥ 8], and total energy intake [kcal/day]), and 4) either perceived stress (for each of coping strategies as a main variable) or all coping strategies (for perceived stress as a main variable) were added to the model in a stepwise manner. A linear trend of the association of each of perceived stress and coping strategies with BMI was assessed by the statistical significant of the corresponding regression coefficient. Gender differences of regression coefficients were tested using the model for both genders combined, with an additional interaction term for each psychosocial variable and gender. Whether the association between each coping strategy and BMI varied by occupation (4 categories) or perceived stress levels (low, medium, and high) was examined in stratified analysis, and was tested by including in the model additional interaction term(s) for either occupation (categorical variable) or perceived stress (ordinal variable) and each coping strategy (ordinal variable). Adjusted means of BMI and their 95% confidence intervals (CIs) by the level of each coping strategy were computed as least square means (i.e., analysis of covariance) by using the GLM procedure with the LSMEANS statement and the OBSMARGINS option in SAS.

Multiple logistic regression analyses were applied to estimate adjusted odds ratios and their 95% CIs of overweight/obesity (BMI ≥ 25.0 kg/m^2^) [30] for each of perceived stress and coping strategies. All *P* values reported are two-tailed, and those values less than 0.05 were considered statistically significant.

## Results

Table 1 shows baseline characteristics of the study subjects with and without overweight/obesity (BMI ≥ 25.0 kg/m^2^) stratified by gender. Men with overweight/obesity were significantly more likely to be younger, use some coping strategies (positive reappraisal and problem solving), have managerial and professional occupations, and sleep shorter, as compared to those without overweight/obesity. Women with overweight/obesity, as compared to those without it, were older, felt more stress, used emotional support seeking less frequently, worked shorter, had lower education, included fewer drinkers, and had higher physical activity levels.

**Table 1:** Characteristics of study subjects (5,063 men and 6,982 women) by body mass index (BMI, < 25 and ≥ 25 kg/m^2^)—Japan, 2005–2007.

Characteristic	Men		*P* ^a^	Women		*P* ^a^
	BMI < 25 (n = 3,487)	BMI ≥ 25 (n = 1,576)		BMI < 25 (n = 5,699)	BMI ≥ 25 (n = 1,283)	
BMI (kg/m^2^)	22.2 (1.9)	27.1 (2.1)	—	21.2 (2.1)	27.5 (2.5)	—
Underweight (< 18.5 kg/m^2^, %)	4.3	—		9.7	—	
Obesity (≥ 30 kg/m^2^, %)	—	9.0		—	13.4	
Age (years)	56.8 (8.2)	56.0 (8.2)	<0.01	55.3 (8.3)	56.9 (7.9)	<0.01
Perceived stress (% of high level^b^)	29.1	29.6	0.43	18.1	20.0	0.02
Coping strategies (% of high level^c^)						
Emotion expression	20.0	21.6	0.18	18.4	17.6	0.51
Emotional support seeking	8.3	9.5	0.17	29.8	26.5	0.02
Positive reappraisal	51.0	54.8	0.01	57.6	61.1	0.33
Problem solving	59.0	63.5	<0.01	54.8	55.1	0.87
Disengagement	30.1	28.3	0.18	36.8	37.3	0.75
Occupation (ISCO-08^d^, %)			<0.01			0.03
0–2	35.2	43.9		17.2	15.8	
3–5	27.9	25.1		30.6	27.9	
6–9	17.8	15.5		9.7	11.4	
Unemployed	19.2	15.5		42.5	44.9	
Working hours ≥9 (%)	37.7	36.3	0.36	29.5	24.0	<0.01
Years of schooling ≥12 (%)	49.4	49.1	0.46	42.2	33.6	<0.01
Current drinker (%)	79.4	79.1	0.80	42.4	37.1	<0.01
Current smoker (%)	36.4	35.5	0.51	8.5	8.1	0.95
Physical activity level^e^	1.45 (0.09)	1.45 (0.09)	0.36	1.46 (0.08)	1.47 (0.09)	0.007
Sleeping hours	6.9 (0.98)	6.7 (1.00)	<0.01	6.5 (0.93)	6.5 (0.98)	0.99
Energy intake (kcal/day)	1935 (340)	1938 (369)	0.81	1514 (227)	1520 (243)	0.42

Data are mean (standard deviation) or percentage.

^a^ P values for the difference by BMI (< 25 and ≥ 25 kg/m^2^) based on t test (for continuous variables) or χ^2^ test (for categorical variables).

^b^ High level represents having felt ‘much’ stress for the last year.

^c^ High level represents the frequency of ‘often’ or ‘very often’ for each coping strategy.

^d^ Based on the International Standard Classification of Occupation 2008 (see the “Methods”). The “unemployed” category includes retired subjects and housewives.

^e^ Calculated as total energy expenditure (kcal/day) divided by basal metabolic rate (kcal/day).

Perceived stress had significant positive correlations with emotion expression, ESS, and problem solving (Table 2). Perceived stress also showed a weak inverse association with disengagement in women, but not in men. As shown in Table 2, higher levels of stress and all coping strategies were associated with younger age in both men and women. Perceived stress and coping strategies were mostly positively correlated with employment, working hours, years of schooling, and alcohol drinking. Only perceived stress and emotion expression showed a positive correlation with smoking. Perceived stress was inversely related to sleeping hours. In men, higher levels of perceived stress and all coping strategies were associated with higher energy intake.

**Table 2:** Spearman’s rank correlation coefficients between psychosocial factors/body mass index and covariates by gender—Japan, 2005–2007.

	Perceived stress	Age	Unemployed^a^	Working hours	Years of schooling	Drinking	Smoking	Physical activity level^b^	Sleeping hours	Energy intake
**MEN** (*n* = 5,063)										
Perceived stress	**-**	**-0.30**	**-0.21**	**0.28**	**0.12**	0.02	**0.06**	-0.01	**-0.14**	**0.03**
Emotion expression	**0.13**	**-0.05**	<0.01	0.01	0.01	**0.06**	**0.04**	<0.01	<0.01	**0.06**
Emotional support seeking	**0.15**	**-0.17**	**-0.09**	**0.09**	**0.06**	**0.03**	0.01	**0.03**	-0.02	**0.08**
Positive reappraisal	-0.02	**-0.06**	**-0.07**	**0.07**	**0.08**	**0.04**	<0.01	0.01	<0.01	**0.03**
Problem solving	**0.10**	**-0.11**	**-0.11**	**0.13**	**0.10**	**0.04**	<0.01	**0.04**	-0.03	**0.04**
Disengagement	0.02	**-0.08**	**-0.05**	**0.04**	**0.04**	<0.01	-0.02	-0.01	**-0.04**	**0.04**
Body mass index	<.001	**-0.04**	**-0.05**	0.02	0.02	**0.03**	-0.01	**0.04**	**-0.08**	<0.01
**WOMEN** (*n* = 6,982)										
Perceived stress	-	**-0.21**	**-0.12**	**0.19**	**0.10**	**0.06**	**0.07**	-0.01	**-0.14**	-0.01
Emotion expression	**0.13**	**-0.11**	<0.01	0.02	**0.05**	**0.04**	**0.03**	**-0.08**	<0.01	**0.03**
Emotional support seeking	**0.18**	**-0.23**	**-0.07**	**0.06**	**0.11**	**0.04**	<0.01	**0.05**	**-0.04**	**0.03**
Positive reappraisal	-0.02	**-0.03**	**-0.05**	**0.05**	**0.08**	**0.03**	<0.01	0.02	**-0.03**	0.02
Problem solving	**0.10**	**-0.08**	**-0.05**	**0.08**	**0.09**	**0.03**	0.01	0.02	**-0.04**	0.01
Disengagement	**-0.03**	**-0.06**	-0.02	0.02	0.01	<0.01	0.01	0.01	<0.01	0.03
Body mass index	**-0.05**	**0.16**	**0.04**	**-0.09**	**-0.09**	**-0.03**	-0.01	**0.07**	0.02	0.01

Categorical variables as described in the Methods were converted to ordinal variables (for working hours, years of schooling, drinking, smoking, sleeping hours) or a dichotomized variable (only for occupation: unemployed versus employed). Age, physical activity level, and energy intake were treated as continuous variables. Bold font represents P < 0.05.

^a^Includes retired subjects and housewives.

^b^Calculated as total energy expenditure (kcal/day) divided by basal metabolic rate (kcal/day).

Table 3 shows the associations of perceived stress and coping strategies with BMI by gender. In men, BMI tended to increase with positive reappraisal and problem solving (*P*_trend_: 0.001 and < 0.001, respectively) and to decrease with disengagement (*P*_trend_ = 0.016). In women, BMI decreased with perceived stress, emotion expression, and ESS (*P*_trend_: < 0.001, 0.016, and < 0.001, respectively). In age-adjusted analysis for men, BMI increased with positive reappraisal and problem solving (*P*_trend_: 0.002 and < 0.001, respectively) and decreased with disengagement (*P*_trend_ = 0. 006). These associations remained significant after adjustment for SES (*P*_trend_: 0.02, 0.006 and 0.01, respectively) (Table 3, Model 1) and lifestyle factors (*P*_trend_: 0.03, 0.009 and 0.001, respectively) (Table 3, Model 2), as well as perceived stress or coping strategies (*P*_trend_: 0.04, 0.007 and < 0.001, respectively) (Table 3, Model 3). In contrast, the univariate associations of perceived stress, emotion expression, and ESS with BMI in women became insignificant after adjustment for age and other factors. In fully adjusted analyses of men and women combined, which correspond to Model 3 in Table 3, there was evidence for gender heterogeneity (i.e., difference between regression coefficients in both genders) in the associations of perceived stress (*P*_interaction_ = 0.004), ESS (*P*_interaction_ = 0.006), positive reappraisal (*P*_interaction_ = 0.003), problem solving (*P*_interaction_ = 0.004), and disengagement (*P*_interaction_ = 0.027) with BMI (data not shown).

**Table 3:** Regression analyses for the associations of perceived stress and coping strategies with body mass index by gender—Japan, 2005–2007.

	Crude		Age-adjusted		Model 1^a^		Model 2^b^		Model 3^c^	
	*β* (SE)	*P*_*trend*_	*β* (SE)	*P*_*trend*_ ^*d*^	*β* (SE)	*P*_*trend*_ ^*d*^	*β* (SE)	*P*_*trend*_ ^*d*^	*β* (SE)	*P*_*trend*_ ^*d*^
Men (*n* = 5,063)										
Perceived stress	0.01 (.06)	0.93	-0.07 (.06)	0.26	-0.09 (.06)	0.13	-0.12 (.06)	0.06	-0.11 (.06)	0.09
Emotion expression	-0.01 (.07)	0.89	-0.02 (.07)	0.75	-0.03 (.07)	0.64	-0.06 (.07)	0.40	-0.04 (.07)	0.53
Emotional support seeking	0.06 (.07)	0.34	0.02 (.06)	0.75	0.01 (.07)	0.90	0.02 (.07)	0.81	0.03 (.07)	0.69
Positive reappraisal	0.20 (.06)	0.001	0.19 (.07)	0.002	0.15 (.06)	0.02	0.13 (.06)	0.03	0.12 (.06)	0.04
Problem solving	0.25 (.06)	< 0.001	0.22 (.07)	< 0.001	0.18 (.07)	0.006	0.17 (.07)	0.009	0.18 (.07)	0.007
Disengagement	-0.15 (.06)	0.016	-0.17 (.06)	0.006	-0.15 (.06)	0.01	-0.20 (.06)	0.001	-0.20 (.06)	< 0.001
Women (*n* = 6,982)										
Perceived stress	-0.21 (.06)	< 0.001	-0.08 (.06)	0.14	-0.04 (.06)	0.44	-0.04 (.06)	0.51	-0.03 (.05)	0.58
Emotion expression	-0.16 (.07)	0.016	-0.08 (.07)	0.22	-0.08 (.07)	0.21	-0.08 (.07)	0.21	-0.08 (.07)	0.22
Emotional support seeking	-0.19 (.05)	< 0.001	0.06 (.06)	0.31	-0.05 (.06)	0.35	-0.03 (.06)	0.56	-0.03 (.06)	0.62
Positive reappraisal	0.07 (.06)	0.25	-0.04 (.07)	0.48	-0.01 (.06)	0.91	-0.02 (.06)	0.72	-0.02 (.06)	0.69
Problem solving	-0.06 (.06)	0.36	0.01 (.06)	0.92	0.05 (.06)	0.41	0.04 (.06)	0.48	0.05 (.06)	0.44
Disengagement	0.02 (.06)	0.79	0.06 (.06)	0.33	0.06 (.06)	0.33	0.06 (.06)	0.27	0.06 (.06)	0.30

Abbreviations: *β*, regression coefficient; SE, standard error. The multiple regression models included body mass index (kg/m^2^) as a dependent variable and each of perceived stress and coping strategies as an ordinal variable (1–3 assigned to low, medium, and high levels, respectively).

The unit of *β* and SE is kg/m^2^.

^a^ Adjusted for age and socio-economic status (occupation, working hours, and years of schooling).

^b^ Adjusted for covariates in Model 1 and lifestyle factors (drinking, smoking, physical activity level, sleeping hours, and energy intake).

^c^ Adjusted for covariates in Model 2 and either perceived stress (for each of coping strategies) or all coping strategies (for perceived stress).

^d^ Represents the statistical significance of *β*.

In additional analyses stratified by the covariates, we noted some differences by occupation in the above associations of problem solving and disengagement with BMI in men (Table 4). The positive association for problem solving and the inverse association for disengagement tended to be clear in the categories 0–2 (armed forces occupations, managers, and professionals) and the categories 6–9 (skilled agricultural, forestry and fishery workers, craft and related trades workers, plant and machine operators, assemblers, and elementary occupations), but not in the categories 3–5 (technicians, associate professionals, clerical support workers, and service and sales workers) and unemployed subjects, although the corresponding interactions were not significant.

**Table 4:** Adjusted means^a^ (and 95% confidence intervals) of body mass index (kg/m^2^) by selected coping strategies and occupation in 5,063 men—Japan, 2005–2007.

Coping strategy	Occupation^b^				*P*_*interaction*_ ^c^
	0–2	3–5	6–9	Unemployed	
Positive reappraisal					0.98
Low	23.8 (23.4–24.3)	23.5 (24.0–23.7)	23.3 (22.8–23.8)	23.3 (22.8–23.8)
Medium	24.0 (23.8–24.2)	23.5 (23.2–23.7)	23.4 (23.0–23.7)	23.2 (22.9–23.5)	
High	24.1 (24.0–24.3)	23.7 (23.5–23.9)	23.4 (23.1–23.7)	23.4 (23.2–23.7)	
	*P*_*trend*_ ^d^ = 0.18	*P*_*trend*_ = 0.27	*P*_*trend*_ = 0.82	*P*_*trend*_ = 0.38	
Problem solving					0.25
Low	23.4 (22.8–24.1)	23.6 (22.9–24.1)	23.3 (22.7–23.8)	23.6 (23.2–24.2)	
Medium	23.9 (23.7–24.2)	23.5 (23.2–23.8)	23.1 (22.8–23.4)	23.0 (22.7–23.4)	
High	24.2 (24.0–24.3)	23.7 (23.4–23.9)	23.5 (23.3–23.9)	23.4 (23.1–23.7)	
	*P*_*trend*_ = 0.02	*P*_*trend*_ = 0.39	*P*_*trend*_ = 0.08	*P*_*trend*_ = 0.91	
Disengagement					0.07
Low	24.4 (24.1–24.7)	23.7 (23.3–24.1)	23.6 (23.2–24.1)	23.6 (23.3–24.1)	
Medium	24.1 (23.9–24.3)	23.5 (23.2–23.7)	23.5 (23.2–23.8)	23.1 (22.9–23.4)	
High	23.8 (23.5–24.0)	23.8 (23.5–24.0)	23.0 (22.6–23.3)	23.2 (22.9–23.6)	
	*P*_*trend*_ = 0.002	*P*_*trend*_ = 0.63	*P*_*trend*_ = 0.009	*P*_*trend*_ = 0.14	

^a^ Adjusted for age, socio-economic status (working hours, and years of schooling), lifestyle factors (alcohol consumption, smoking, physical activity level, sleeping hours, and energy intake), and perceived stress.

^b^ Based on the International Standard Classification of Occupation 2008 as follows: 0–2 (armed forces occupations, managers, and professionals); 3–5 (technicians, associate professionals, clerical support workers, and service and sales workers); 6–9 (skilled agricultural, forestry and fishery workers, craft and related trades workers, plant and machine operators, assemblers, and elementary occupations); and unemployed subjects including retired persons and housewives.

^c^ Interaction between each coping strategy (ordinal) and occupation (categorical) on body mass index (continuous).

^d^ Based on multiple linear regression analysis including in the model an ordinal variable for each coping strategy (1–3 assigned to low, medium, and high levels, respectively).

**Fig 1:**
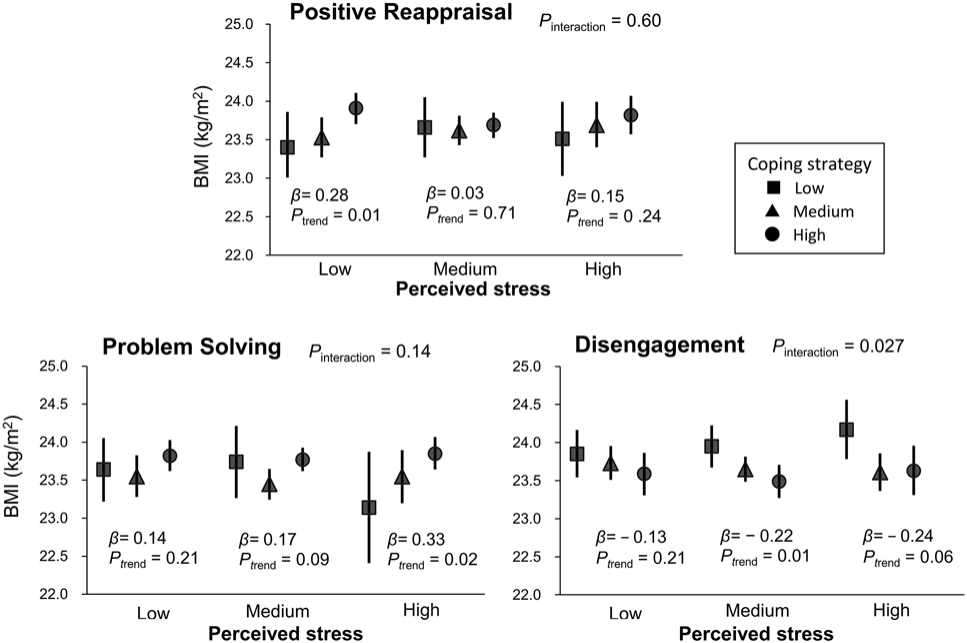
Adjusted means of body mass index (BMI) by levels of selected coping strategies and by perceived stress levels in men. *β* represent regression coefficients in kg/m^2^. Adjustment was made for age, socio-economic status (occupation, working hours, and years of schooling), and lifestyle factors (drinking, smoking, physical activity level, sleeping hours, and energy intake). P values for trend were estimated from multiple linear regression analysis including in the model an ordinal variable (1–3 assigned to low, medium, and high levels, respectively) for each coping strategy. Symbols show means and error bars represent 95% confidential intervals.

We further examined if the above associations of positive reappraisal, problem solving, and disengagement with BMI in men were modified by perceive stress levels (Fig. 1). The positive association between problem solving and BMI was more evident in higher levels of stress (*β* = 0.14 with *P*_trend_ = 0.21 in low, *β* = 0.17 with *P*_trend_ = 0.09 in medium, and *β* = 0.33 with *P*_trend_ = 0.02 in high) although the corresponding interaction was not significant (*P*_interaction_ = 0.14). In contrast, disengagement revealed a clearer inverse association with BMI in higher levels of stress (*β* = −0.13 with *P*_trend_ = 0.21 in low, *β* = −0.22 with *P*_trend_ = 0.01 in medium, and *β* = −0.24 with *P*_trend_ = 0.06 in high) with a significant interaction (*P*_interaction_ = 0.027).

Finally, the associations of perceived stress and coping strategies with overweight/obesity (BMI ≥ 25.0kg/m^2^) were assessed by using multiple logistic regression analyses (Table 5). We observed significant associations of positive appraisal (odds ratio for high vs. low level = 1.25, 95% CI 1.02–1.54; *P*_trend_ = 0.03) and disengagement (odds ratio 0.79, 95% CI 0.67–0.95; *P*_trend_ = 0.01) with overweight/obesity in men. The adjusted odds ratios for problem solving in men were found to be statistically insignificant (odds ratio for high vs. low level = 1.15, 95% CI 0.91–1.46) although the test for linear trend showed statistical significance (*P*_trend_ = 0.03).

**Table 5:** Adjusted odds ratios^a^ (and 95% confidence intervals) of overweight/obesity (body mass index ≥ 25 kg/m^2^) for each of perceived stress and coping strategies by gender—Japan, 2005–2007.

	Level of stress or coping strategy			
	Low	Medium	High	*P*_*trend*_ ^b^
Men (*n* = 5,063)				
Perceived stress	1 (reference)	0.92 (0.79–1.07)	0.93 (0.77–1.11)	0.40
Emotion expression	1 (reference)	0.86 (0.73–1.01)	0.92 (0.75–1.13)	0.47
Emotional support seeking	1 (reference)	0.96 (0.84–1.09)	1.08 (0.86–1.35)	0.90
Positive reappraisal	1 (reference)	1.16 (0.93–1.44)	1.25 (1.02–1.54)	0.03
Problem solving	1 (reference)	0.97 (0.76–1.24)	1.15 (0.91–1.46)	0.03
Disengagement	1 (reference)	0.86 (0.73–0.99)	0.79 (0.67–0.95)	0.01
Women (*n* = 6,982)				
Perceived stress	1 (reference)	1.00 (0.84–1.19)	1.06 (0.88–1.28)	0.51
Emotion expression	1 (reference)	0.90 (0.76–1.07)	0.89 (0.72–1.10)	0.30
Emotional support seeking	1 (reference)	1.00 (0.85–1.18)	0.95 (0.79–1.14)	0.53
Positive reappraisal	1 (reference)	0.96 (0.75–1.22)	0.93 (0.74–1.19)	0.54
Problem solving	1 (reference)	0.91 (0.72–1.15)	0.99 (0.79–1.24)	0.60
Disengagement	1 (reference)	0.87 (0.72–1.06)	0.92 (0.76–1.13)	0.77

^a^Adjusted for age, socio-economic status (occupation, working hours, and years of schooling), lifestyle factors (drinking, smoking, physical activity level, sleeping hours, and energy intake), and either perceived stress (for each of coping strategies) or all coping strategies (for perceived stress).

^b^P values for trend were estimated from multiple linear regression analysis including in the model an ordinal variable (1–3 assigned to low, medium, and high levels, respectively) for each of perceived stress and coping strategies.

## Discussion

In the present study, perceived stress was not significantly associated with BMI in either men or women. Instead, we observed that, in men, positive reappraisal and problem solving had a positive association with BMI, and disengagement revealed an inverse association with it even after controlling for perceived stress and other covariates. The associations of problem solving and disengagement with BMI tended to be modified by perceived stress levels, as indicated by a clearer association at higher levels of perceived stress. To examine the clinical relevance of the above findings, we also carried out multiple logistic regression analyses with overweight/obesity as a dependent variable and found that the high level of either positive reappraisal or disengagement was significantly and independently associated with overweight/obesity in men (odds ratios for high vs. low level: 1.25 and 0.79, respectively).

The overall evidence appears to suggest a weak positive association between psychosocial stress and adiposity [4]. Although we could not find any significant positive association between perceived stress and BMI in either gender, this finding is not necessarily inconsistent with previous results as reviewed in a recent meta-analysis showing that null results were most prevalent (69%) [4]. If the true association between psychosocial and adiposity is so weak (combined r = 0.014) [4], this study of a relatively large sample size would still be underpowered to detect such a weak association. Otherwise, some differences between this and other studies in characteristics of study subjects (e.g., occupation and prevalence of mental illness and obesity) [7,8] and measurements of stress (general perceived stress in this study vs. stress related to job, income, and life events in other studies [5–7]) may partly account for the above inconsistency.

Regarding coping strategies, only one study reported that young and middle-aged African American women showed an inverse association between confrontive coping and BMI [14]. However, that study did not control for age. Although we observed similar inverse associations between some coping strategies (i.e., emotion expression and ESS) and BMI in women, these associations disappeared after adjustment for age, suggesting that potential confounding by age might explain the discrepancy between the previous [14] and present studies. The difference in age range of study subjects (18–45 years in the previous study [14] vs. 40–69 years in this study) might also be partly responsible for such a discrepancy.

Psychosocial stress has been suspected to develop obesity partly through its associations with sleeping status [31], eating behaviors [32], and poor physical activity [33], and by similar mechanisms, coping strategies might be connected with obesity. In fact, we found weak positive relations of coping strategies to drinking and energy intake (Table 2). However our findings remained unchanged even after controlling for lifestyle factors (drinking, smoking, physical activity level, sleeping hours, and energy intake) as well as SES and psychosocial variables, so coping strategies might be involved in increased BMI by other mechanisms. Cortisol, a major stress hormone, promotes the accumulation of triglycerides in adipocytes [34], thereby leading to obesity. Because a previous study reported that lower levels of social support seeking and problem engagement were related to higher levels of cortisol over the day [13], we hypothesized that lower coping strategies might be associated with higher BMI. Contrary to this hypothesis, positive reappraisal and problem solving had a positive association with BMI in men although disengagement revealed a male-specific inverse association. Due to the paucity of relevant data on cortisol and coping strategies, this issue warrants further studies.

Positive reappraisal and disengagement are both reinterpreting the meaning of negative stimuli, with a resulting reduction in emotional responses. However, positive reappraisal is changing one’s own emotional reaction whereas disengagement represents avoidant coping [35], indicating some difference between both strategies. In fact, their mutual correlation was very weak in our study (r = 0.022 for men and 0.018 for women) [19]. A previous study reported that lower systolic blood pressure was associated with avoidant coping, but not with positive reappraisal [36], suggesting that these strategies can lead to different health outcomes, as indicated by this study.

We noted gender heterogeneity in the association between coping strategies and BMI; positive reappraisal, problem solving, and disengagement had a significant association with BMI in men only. In a meta-analysis of longitudinal studies regarding psychosocial stress and adiposity, positive effects in men tended to be stronger than those in women [4]. Several studies also reported that the positive association of work stress as evaluated by the effort-reward-imbalance model and over-commitment with cortisol levels was more pronounced in men [37,38]. Although these findings are not directly relevant to coping strategies, some gender-dependent responses of cortisol and other unknown mediators with coping strategies triggered by psychosocial stress might contribute to the male-specific associations between coping strategies and BMI in this study.

In stratified analyses, we found some differences by occupation in the above associations of problem solving and disengagement with BMI in men although the corresponding interactions were not significant. These associations were evident in professionals (categories 0–2) and blue collar workers (categories 6–9), but not in white collar workers (categories 3–5) and unemployed subjects. We currently have no plausible explanations for this finding, and chance could be responsible. Unemployed subjects included retired and jobless people as well as housewives, who may have different significance in coping strategies. Unfortunately, we could not address the significance of heterogeneity in unemployed subjects due to the lack of relevant data.

It was also notable that the associations of problem solving and disengagement with BMI were modified by the level of perceived stress. To our knowledge, no studies have been conducted on this issue, but a few studies demonstrated that the relations of some coping strategies to high-sensitivity C-reactive protein [19] and depression [39] were dependent on the level of psychosocial stress. In this study, the positive association between problem solving and BMI and the inverse association between disengagement and BMI were more evident in higher levels of stress. This finding suggests that these coping strategies may play an important role in the increase or decrease of BMI, particularly among individuals with high stress levels. The lack of information on coping strategies in previous studies might have obscured true associations to some extent.

The strength of our study includes the large sample size and the detailed measures of SES and lifestyle factors as covariates. Meanwhile, several limitations should be mentioned. First, because we could not explicitly exclude subjects with mental illness, our findings may have been affected by some connection of perceived stress or coping strategies with relatively common mental illness (e.g., depression and anxiety disorders) [40]. Although we could not assess the prevalence of such mental illness, our study included 46 subjects (0.4%) with self-reported treatment for depression and 140 subjects (1.2%) with self-reported medication of antipsychotics, antidepressants, and/or anxiolytics. Both prevalences were lower than those reported in Japan [41,42], suggesting that those mental illnesses may have been underrepresented in our study subjects. Second, we did not obtain information on detailed eating behaviors (e.g., eating binges [43], midnight snack intake, and the time of eating, especially of dinner). Thus, we could not exclude the possibility that such eating behaviors might have mediated the association between some coping strategy and BMI although we excluded subjects with an extreme energy intake (> 4000 kcal/day) and adjusted for energy intake. In particular, eating binges is one of the important stress coping behaviors, which is observed more frequently women [43]. In our female subjects, however, the lack of any significant associations between coping strategies and BMI after adjustment for age only (Table 3) suggests the absence of important mediators including eating binges, at least in this study. Third, perceived stress and coping strategies were evaluated by using simple questions. For coping strategies, the poor to fair reproducibility (weighted *κ* 0.30–0.55) [19,44] may have weakened true associations. Fourth, our results on coping strategies and BMI may not be regarded as causal relationships due to the cross-sectional design. Finally, the study subjects consisted of only the Japanese, so our results may not be generalized to other ethnicities with different psychosocial backgrounds.

In conclusion, we found that lower levels of disengagement and higher levels of positive reappraisal and problem solving were independent predictors of increased BMI and overweight/obesity in Japanese middle-aged and older men. Our findings suggest that some coping strategies may have an important role in developing obesity, so we recommend that future studies should assess the risk of obesity related to psychosocial stress, with taking into account the influence of coping strategies.
